# Do small-sided games prepare players for the worst-case scenarios of match play in elite young soccer players?

**DOI:** 10.5114/biolsport.2024.127389

**Published:** 2023-07-19

**Authors:** Vicente de Dios-Álvarez, Julen Castellano, Alexis Padrón-Cabo, Ezequiel Rey

**Affiliations:** 1 Faculty of Education and Sports Sciences. University of Vigo, Pontevedra, Spain; 2 Faculty of Education and Sport, University of the Country Basque (UPH/EHU). Vitoria-Gasteiz, Spain; 3 Methodology Department, Real Club Celta, Vigo, Pontevedra, Spain; 4 Department of Physical and Sports Education, Faculty of Sports Sciences and Physical Education, University of A Coruña, A Coruña, Spain

**Keywords:** Performance analysis, Match peak, Physical demands, SSG, Team sport

## Abstract

The aim of the study was to determine whether the physical performance of young soccer player during various small sided games (SSGs) underloads, replicates or overloads the requirements of the worst-case scenarios (WCS) during match play. A total of 521 SSGs’ individual observations and 15 different formats of SSGs with different areas per player (ApP) (ApP100: < 100; ApP200: ranged from 101 to 200; ApP300: > 201, all in m^2^ · player^-1^) were taken into consideration. Whole (90-min average; OM) and 15-, 5- and 1-min worst-case scenarios (WCS15, WCS5 and WCS1, respectively) were analysed. Total distance covered relative (m · min^-1^) (TDCR), high-speed distance relative (m · min^-1^) (HSDR), very high-speed distance relative (m · min^-1^) (VHSDR) and sprint distance relative (m · min^-1^) (SDR), player load relative (PLR) and both total (ACCR) and high intensity relative accelerations (n · min^-1^) (ACCHR) were collected. All external load measures analysed were significantly higher in WCS1 compared to WCS of longer duration and SSGs with different ApP (p < 0.001). The analysis demonstrated interactions between game type and player positions (p < 0.001) for TDCR, VHSDR, PLR and ACCHR. The SSG formats did not sufficiently stimulate the WCS for locomotor demands (VHSDR and SDR). SSGs played on an ApP100 overestimated the mechanical values compared to WCS15 and WCS5. The analysed SSG formats did not sufficiently stimulate players to cope with all external load demands that occurred during WCS1. This study provides useful information for practitioners on the heightened impact of different SSG formats on external load in relation to the WCS of competitive match play.

## INTRODUCTION

Small sided games (SSGs) are currently one of the most common tasks used to enhance players’ soccer-specific technical, tactical, and physical skills [1]. In fact, SSGs allow players to replicate specific physical, technical, and tactical defensive and offensive behaviours without having to repeat mechanical movements [2]. According to previous scientific literature, the physical performance and the development of fatigue in SSGs vary depending upon several variables [1, 2]. Two of the most often used constraints in designing SSGs are the number of players and the pitch size [3]. When both variables are combined, the area per player (ApP) is obtained. It is a key concept during this type of task and it is defined as the theoretical pitch area that corresponds to each player; it is determined as the total pitch area divided by the number of players on the pitch [4]. From a practical perspective, previous research revealed that large areas lead to an increase in total distance, total distance per minute, distance at different intensities, and sprint frequency [4, 5]. However, controversy exists regarding accelerations and decelerations. It seems that with smaller ApP increases in acceleration and deceleration are reported [6].

Monitoring training and match load can provide a scientific explanation for changes in performance. Load monitoring could be fundamental to reducing the risk of injury, optimizing performance, and avoiding non-functional overtraining [7, 8]. In addition, professional soccer players have increased the high-speed distance (HSD) and sprint distance (SD) performed in match play over time, highlighting the importance of monitoring these factors during training, specifically during training tasks [9]. Hence quantifying training load (TL) is important to obtain overall knowledge of how the training sessions or tasks such as SSGs differ from the official match (OM) demands [10]. Previous research analysed the differences between SSGs and friendly matches [11]; these authors concluded that high-intensity distance during friendly matches was greater than during SSGs. However, the global indicator of workload (work ratio and player load) was higher during SSGs than in friendly matches. More recently, Pinheiro et al. [12] stated that with professional soccer players the total distance per minute was higher during friendly matches than during different formats of SSGs. Additionally, no differences were found between friendly matches and SSGs considering mechanical values (number and average distance of accelerations). Another group of authors compared different SSGs with OMs [13] and found that only large-sided games (9vs9; 194 m^2^ · player^-1^) simulated the official full match more accurately than other sided games in terms of sprinting. Medium and SSGs were more intense than full matches considering mechanical values. However, these studies took into account physical performance variables for whole matches. Whilst reporting half or whole-match activities is useful to help understand overall physical loading, such data do not reflect the stochastic nature of soccer match-play [14]. Therefore, considering the most intense periods of the competition could be valuable when managing the TL.

The worst-case scenarios (WCS) are defined as the periods of maximum physical output throughout the match [14]. If the objective of training is to replicate or overload the movement demands reported during the competition, the use of WCS could be key to optimizing performance and adequately preparing the player for the matches. Hence, elucidating the demands associated with the WCS and comparing them with one of the most used tasks in training sessions (i.e., SSGs) may be useful when developing and scheduling specific training sessions. Although there are studies analysing the differences between SSGs and official competitions [6, 13], to the authors’ knowledge there is a gap in the literature examining the differences between SSGs and WCS during soccer competitions. Previous research [15, 16, 17] analysed the differences between SSGs and WCS. Nevertheless, only one previous study reported these differences considering young soccer players [17]. This information may be useful for developing soccer-specific training programmes designed to condition youth players to cope with potentially decisive periods of matches. The first group of authors with senior

Norwegian professional players indicated that HSD during 4vs4 and 6vs6 was 78% and 86% lower than in the peak period of match play, showing that this type of SSGs could underestimate high-intensity activities reported during the WCS. It seems that the distance, HSD and distance covered when sprinting are the variables that have the lowest percentage of most demanding passages while performing the game formats studied, especially in the smallest formats of training games [16]. Meanwhile, with elite young players, Sydney et al. [17] mentioned that no SSGs of different sizes were found to replicate the peak of total distance and HSD during match-play. However, these authors only considered two locomotor variables (total distance and HSD). In agreement with Martin-Garcia et al. [16] we believe that using more time windows (e.g., 1, 5, and 15 minutes) would have made it possible to explore in greater depth how the values relating to the competition percentage vary. In consequence, more research in young players using more WCS time windows and considering more variables is needed.

Hence, the objective of this study was to determine whether the physical performance of young soccer players during various SSGs underloads, replicates or overloads the requirements of the worst-case scenarios during match play. The conclusions of the present study would make it possible to manage the ApP used during SSG to underload, replicate or overload the requirements of the worst-case scenarios during match play and plan the whole-session training load during practice.

## MATERIALS AND METHODS

### Subjects

The data for this study were collected from players belonging to an elite Spanish soccer academy (*n*= 31; age = 17.0 ± 1.3 years; height = 173.7 ± 6.0 cm; body mass = 66.9 ± 7.1 kg and body fat percentage = 10.6 ± 1.0%). Goalkeepers were excluded from the data collection. The club’s medical staff certified the health status of each player. Injured players diagnosed by medical services or players involved in rehabilitation training sessions were also excluded from data collection. Participants were categorized according to playing positions as directed by the head coach. Playing positions were CD = Central Defender (n = 11 and 225 observations), FB = Fullback (n = 5 and 159 observations), MD = Midfielder (n = 3 and 114 observations), WM = Wide Midfielder (n = 8 and 217 observations) and FW = Forward (n = 4 and 99 observations) (Table 1). A consent letter was obtained from the club agreeing with the procedures. The local Ethics Committee (E1621871) approved the study and it was performed in accordance with the principles of the Declaration of Helsinki. Since data used in the present study were acquired as part of players’ routine monitoring, informed consent was not required [18].

**TABLE 1:** Number of observations of each SSGs, OM and WCS according to player positions.

Position	ApP100	ApP200	ApP300	OM	WCS15	WCS5	WCS1	Total
**CD**	62	27	53	18	22	22	21	225
**FB**	37	18	33	14	19	19	19	159
**MF**	32	13	16	11	14	14	14	114
**WM**	39	26	56	21	25	25	25	217
**FW**	18	9	21	9	14	14	14	99
**Total**	188	93	179	73	94	94	93	814

Note: WCS15 (worst case scenario of 15 minute of duration); WCS5 (worst case scenario of 5 minute of duration); WCS1 (worst case scenario of 1 minute of duration); ApP100 (Area per player < 100 m^2^ · player ^-1^); ApP200 (Area per player between 100 and 200 m^2^ · player^-1^); ApP300 (Area per player > 200 m^2^ · player^-1^); OM (official match); CD (Central Defender); FB (Fullback); MF (Midfielders); WM (Wide Midfielder); FW (Forward).

### Training contents and matches

A total of 521 SSGs’ individual observations were undertaken (average per player: 26.3 (± 17.5) and all SSGs were grouped according to the ApP. SSGs ranged from 10 vs 10 to 3 vs 3 and ApP ranged from 60 to 332 m^2^ · player^-1^ (with 24 different ApP). The SSGs’ training formats are described in Table 2. The ApP was obtained as the total pitch area divided by the number of players on the pitch [19]. Goalkeepers were present for each game type although they were excluded in the calculations when determining the relative pitch area per player (m^2^). Small, medium and large-sided games were all abbreviated as SSGs and specified by ApP (ApP100: < 100 m^2^ ·player^-1^; ApP200: ranged from 101 to 200 m^2^ · player^-1^; ApP300: ranged from 201 to 350 m^2^ · player^-1^). The ApP was used as a discrete variable to compare the physical demands of SSGs with different ApP with those of WCS during OM. The SSGs were performed under the supervision and motivation of several coaches to maintain a high work ratio. In addition, a ball was immediately made available by replacement when it went out of play. In SSGs, the corners were replaced by a ball in game from the goalkeeper (except for 10 vs 10, where corners were performed). Coach feedback was present during each SSG and players were instructed to pressure the opposition as much as possible [17].

**TABLE 2:** Pitch size during small sided games.

N° players	length:width (m)	width:length ratio	m^2^	m^2.^player^-1^	Goalkeeper	Floater	Duration Range (min)	Bouts Range
10 vs 10	104 × 62	0.59	6448	322	Yes	0	8-30 min	2-4
	75 × 62	0.86	4650	232				
	70 × 62	0.88	4340	217				
	67 × 62	0.92	4154	208				
	62 × 65	1.04	4030	202				
	60 × 62	1.03	3720	186				
	58 × 64	1.10	3712	185				

9 vs 9	62 × 55	0.88	3410	179	No	1	8-15 min	1-2
	60 × 40	0.66	2400	126				

8 vs 8	61 × 62	1.01	3782	236	Yes	0	10-11 min	1-2
	54 × 50	0.92	2700	168				
	65 × 62	0.95	4030	237	Yes	1		

6 vs 6	35 × 32	0.91	1120	93	Yes	0	4-6 min	2-4

5 vs 5	30 × 32	1.06	960	96	Yes	0	2-5 min	2-4
	30 × 26	0.86	780	78				
	27 × 25	0.92	675	68				

4 vs 4	30 × 26	0.86	780	98	Yes	0	3-4 min	2-4

3 vs 3	18 × 22	1.22	396	66	Yes	0	2-3 min	3
	21 × 18	0.85	378	54	Yes	1		

A total of 11 OM and 752 records (CD = 176, FB = 152, MF = 112, WM = 200 and FW = 112) were considered to analyse the WCS of match play. Only players who started the match and completed the whole OM were included. Each match was ninety minutes in duration, separated by two forty-five-minute halves, with any additional time determined by the match referee. All matches were played under the same competition rules, limiting each team to three substitutions and a fifteen-minute break for half-time. Matches were preceded by a twenty-minute standardized warm-up consisting of dynamic stretching, 20 m and 30 m maximal sprint efforts, short and long passing, and possession play (4 vs 4 plus 2 floaters). Matches were played on official fields (104 × 62 m, length × width, respectively). Both mean values and peak 15-, 5-, and 1-minute values were calculated. Hence, for each player and for each variable, the most intense phases of 15-, 5-, and 1-minute duration were calculated using rolling average methods [20].

### External load variables

The running variables were obtained from the Global Positioning System (GPS). All external load measures were normalized as relative distance covered in one minute (m · min^-1^) or the number of accelerations in one minute (n · min^-1^) [21]. Consistent with a previous study [22] that utilised similar thresholds, the movement demands were reported as total distance covered relative (m · min^-1^) (TDCR), high-speed distance relative (HSDR) (> 18 km· h^-1^), very high-speed distance (m · min^-1^) relative (VHSDR) (> 21 km · h^-1^) and sprint (> 25 km · h^-1^) distance m) relative (SDR). The total number (per minute) of accelerations (ACCR) and the total number of high-intensity accelerations (ACCHR) (> 3 m · s^-2^) were also gathered [5, 20]. Moreover, a global load indicator was included as a variable: player load per minute (PLR), which is a measure based on the tri-axial accelerometer measures and may serve as a complementary tool for measuring the load from activities misrepresented by time-motion analysis [15].

### Procedures

The participants undertook their traditional weekly training routine. All training sessions were performed on artificial pitches and all training sessions were scheduled at the same time (16:30-18:45). During both training sessions and OM, players’ movements were recorded using a portable 10 Hz GPS device that also incorporates a 400 Hz tri-axial accelerometer (Playertek, Dundalk, Ireland). Acceleration activity was measured as a change in speed for a minimum period of 0.5 seconds with acceleration at least of 2 m · s^-2^. These GPS devices seem to be valid and reliable for use in team sports [23] and they were used previously in soccer research [24]. The GPS device was attached to the upper back of each player by means of a special harness, and according to the manufacturer’s instructions, all GPS units were activated 10 minutes before the training sessions or OM began.

### Statistical analysis

Descriptive statistics (mean ± SD and confidence intervals) are reported for all variables. The data were tested for normality using quantile-quantile plots (Q-Q plots) and the Kolmogorov-Smirnov test of normality. Linear mixed models were performed to analyse the differences between SSG formats (ApP100, ApP200 and ApP300), mean OM and WCS with different durations (WCS15, WCS5, and WCS1). The players’ identity was modelled as a random effect to take into account the repeated measurements. Effect size (ES) was established using Cohen’s *d*. Concretely, the ES was calculated according to the formula *d* = (M_2_ – M_1_/SD_pooled_), where M_1_ and M_2_ are the means of the two groups and SD_pooled_ is the square root of the weighted average SD. According to Cohen [25], ES were classified as *trivial* (< 0.1), *small* (0.1-0.3), *moderate* (0.3-0.5), *large* (0.5-0-7) and *very large* (> 0.7). Data analysis was conducted using SPSS Statistics (IBM Corp. Released 2017. IBM SPSS Statistics for Macintosh, Version 25.0. Armonk, NY: IBM Corp.) software with a significance value set at p < 0.05.

## RESULTS

Descriptive statistics for selected physical performance variables and differences between formats (different WCS, OM, ApP100, ApP200, and ApP300) are shown in Table 3. All external load measures analysed (TDCR, HSDR, VHSDR, SDR, PLR, and ACCHR) were significantly higher in WCS1 compared to WCS of longer duration and SSGs with different ApP. Specifically, when TDCR is analysed, it was found that all WCS were significantly higher than the SSG formats (p < 0.001; ES: 0.8–5.3). Additionally, ApP300 elicited significantly higher TDCR values compared to ApP100 (p < 0.05; ES: 0.62). Considering HSDR and VHSDR, WCS with different duration (WCS1, WCS5 and WCS15) were significantly greater than all SSG formats (p < 0.001; ES: 1.3–5.3) Moreover, SSGs with ApP300 and ApP200 had significantly higher HSDR (p < 0.05; ES: 1.6 and ES: 1.1, respectively) and VHSDR (p < 0.05; ES: 1.7 and ES: 0.9, respectively) values than ApP100. SDR was significantly higher in all WCS compared to all SSG formats analysed (p < 0.01; ES: 2.9–1.4). However, no differences were found between SSGs taking into consideration SDR values. Regarding mechanical measures (PLR, ACCR, and ACCHR), WCS1 was significantly greater compared to both worst-case scenarios of longer duration and SSGs with different ApP (p < 0.001). Significantly higher PLR values were found in WCS5 (p < 0.05; ES: 1.8-1.1) and WCS15 (p < 0.05; ES: 0.3–0.8) compared to the SSGs analysed. Furthermore, ApP200 and ApP300 showed lower PLR values than ApP100 (p < 0.05; ES: 0.4 and ES: 0.5, respectively). Taking into consideration acceleration values, both total and high-intensity accelerations, for WCS5 and ApP100 significantly higher ACCHR was found than for WCS15 (ES: 1.5 and ES: 1.3, respectively), ApP200 (ES: 1.8 and ES: 1.5, respectively) and ApP300 (ES: 2.3 and ES: 1.7, respectively) (Table 3).

**TABLE 3:** Physical performance variables (mean, standard deviation, and range) for three worst case scenarios (WCS15, WCS5 and WCS1), OM and different types of small-sided games (ApP100, ApP200 and ApP300).

	TDCR	HSDR	VHSDR	SDR	ACCR	ACCHR	PLR
WCS15	129.5 ± 13.0	18.4 ± 5.1	10.3 ± 4.0	4.2 ± 2.5	7.0 ± 1.1	1.6 ± 0.4	5.5 ± 0.6
	(126.8–132.2)	(17.3–19.4)	(9.4–11.1)	(3.7–4.7)	(6.8–7.2)	(1.5–1.7)	(5.4–5.7)
WCS5	143.3 ± 10.4	27.3 ± 6.9	16.8 ± 5.7	8.4 ± 4.2	8.3 ± 1.1	2.2 ± 0.4	6.1 ± 0.5
	(141.2–145.5)	(25.8–28.6)	(15.7–18.0)	(7.5–9.2)	(8.1–8.5)	(2.1–2.3)	(6.0–6.2)
WCS1	194.1 ± 14.1	70.7 ± 17.3	50.2 ± 15.3	30.5 ± 14.4	12.9 ± 1.4	4.9 ± 0.9	8.1 ± 0.6
	(191.2–197.1)	(67.2–74.3)	(47.0–53.3)	(27.5–33.4)	(12.6–13.2)	(4.7–5.1)	(7.9–8.2)
OM	116.0 ± 9.5	12.9 ± 4.0	6.4 ± 2.7	2.1 ± 1.3	5.9 ± 0.8	1.2 ± 0.3	4.9 ± 0.5
	(113.5–117.9)	(11.9–13.8)	(5.7–7.0)	(1.7–2.4)	(5.7–6.1)	(1.2–1.3)	(4.8–5.1)
ApP100	103.6 ± 19.6	4.3 ± 3.7	1.1 ± 1.7	0.1 ± 0.6	10.0 ± 2.7	2.8 ± 1.2	5.3 ± 0.9
	(100.7–106.4)	(3.8–4.9)	(0.8–1.3)	(0.1–0.2)	(9.6–10.4)	(2.6–2.9)	(5.2–5.5)
ApP200	111.2 ± 18.3	8.8 ± 4.5	3.2 ± 2.5	0.6 ± 1.0	7.2 ± 1.5	1.5 ± 0.4	5.0 ± 0.8
	(107.4–115.0)	(7.8–9.7)	(2.7–3.7)	(0.4–0.8)	(6.9–7.5)	(1.4–1.6)	(4.8–5.1)
ApP300	115.4 ± 18.3	11.6 ± 4.9	5.3 ± 2.9	1.3 ± 1.5	6.6 ± 1.2	1.3 ± 0.4	4.9 ± 0.8
	(112.7–118.1)	(10.9–12.3)	(4.8–5.7)	(1.1–1.5)	(6.4–6.7)	(1.3–1.4)	(4.8–5.1)
Statistical differences (p < 0.05)	WCS1 > WCS5, WCS15, OM, ApP100, ApP200, ApP300 WCS5 > WCS15, OM, ApP100, ApP200, ApP300 WCS15 > OM, ApP100, ApP200, ApP300 OM, ApP300, ApP200 > ApP100	WCS1 > WCS5, WCS15, OM, ApP100, ApP200, ApP300 WCS5 > WCS15, OM, ApP100, ApP200, ApP300 WCS15 > OM, ApP100, ApP200, ApP300 OM, ApP300 > ApP100, ApP200 ApP200 > ApP100	WCS1 > WCS5, WCS15, OM, ApP100 ApP200, ApP300 WCS5 > WCS15, OM, ApP100, ApP200, ApP300 WCS15 > OM, ApP100, ApP200, ApP300 OM > ApP200, ApP100 ApP300 > ApP100	WCS1 > WCS5, WCS15, OM, ApP100, ApP200, ApP300 WCS5 > WCS15, OM, ApP100, ApP200, ApP300 WCS15 > ApP100, ApP200, ApP300	WCS1 > WCS5, WCS15, OM, ApP100, ApP200, ApP300 WCS5 > WCS15, OM, ApP200, ApP300 ApP200 > OM ApP100 > WCS5, WCS15, OM, ApP200, ApP300	WCS1 > WCS5, WCS15, OM, ApP100, ApP200, ApP300 WCS5 > WCS15, OM, ApP200, ApP300 WCS15 > OM, ApP300 ApP100 > WCS5, WCS15, OM, ApP200, ApP300	WCS1 > WCS5, WCS15, OM, ApP100, ApP200, ApP300 WCS5 > WCS15, OM, ApP100, ApP200, ApP300 WCS15 > OM, ApP200, ApP300 ApP100 > ApP200, ApP300

Note: TDCR (total distance covered relative); HSDR (high speed distance relative); VHSDR (very high speed distance relative); SDR (sprint distance relative); PLR (player load relative); ACCR (the number of total accelerations relative); ACCHR (the number of high accelerations relative); WCS15 (worst case scenario of 15 minute of duration); WCS5 (worst case scenario of 5 minute of duration); WCS1 (worst case scenario of 1 minute of duration); ApP100 (Area per player < 100 m^2^ · player^-1^); ApP200 (Area per player between 100 and 200 m^2^ · player^-1^); ApP300 (Area per player > 200 m^2^ · player^-1^); OM (official match).

**FIG. 1:**
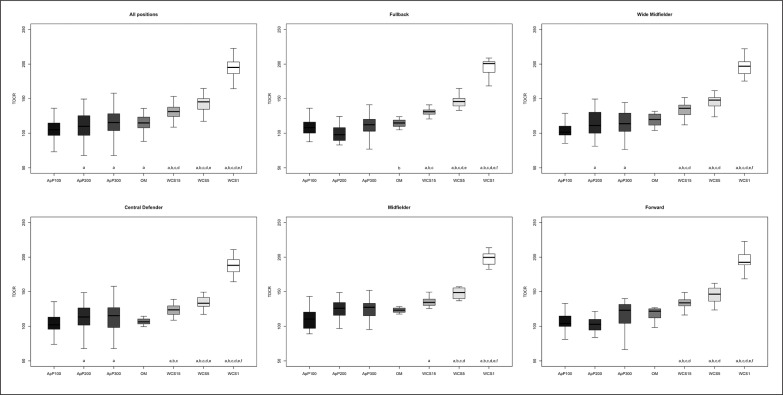
Significant differences between different WCS and various sided games considering total distance covered relative (TDCR); a: significant differences compared to ApP100; b: significant differences compared to ApP200; c: significant differences compared to ApP300; d: significant differences compared to OM; e significant differences compared to WCS15; f: significant differences compared to WCS5.

**FIG. 2:**
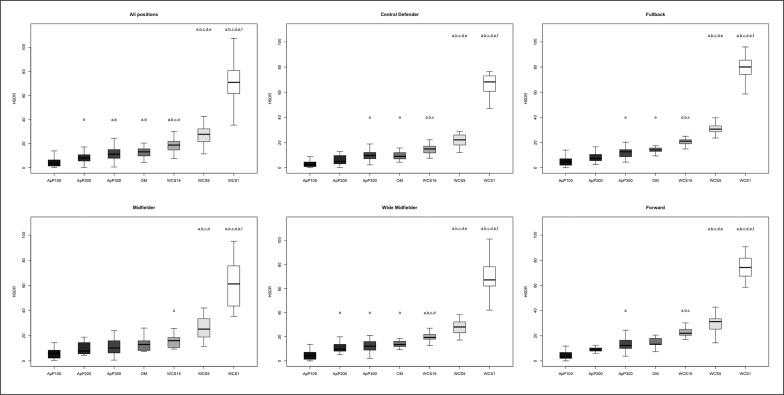
Significant differences between different WCS and various sided games considering high-speed distance relative (HSDR); a: significant differences compared to ApP100; b: significant differences compared to ApP200; c: significant differences compared to ApP300; d: significant differences compared to OM; e significant differences compared to WCS15; f: significant differences compared to WCS5.

**FIG. 3:**
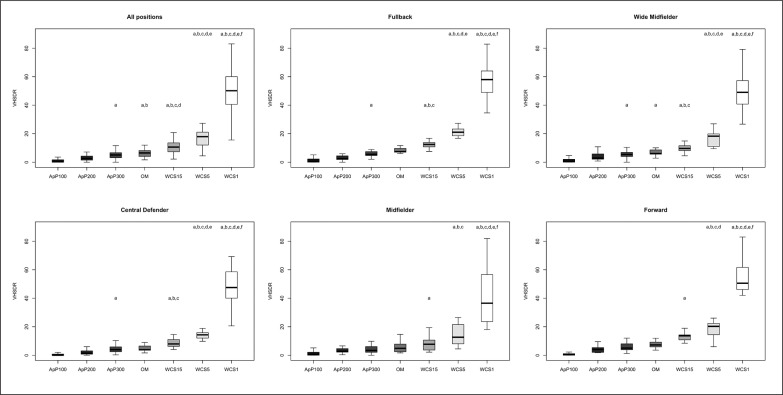
Significant differences between different WCS and various sided games considering very high-speed distance relative (VHSDR); a: significant differences compared to ApP100; b: significant differences compared to ApP200; c: significant differences compared to ApP300; d: significant differences compared to OM; e significant differences compared to WCS15; f: significant differences compared to WCS5.

**FIG. 4:**
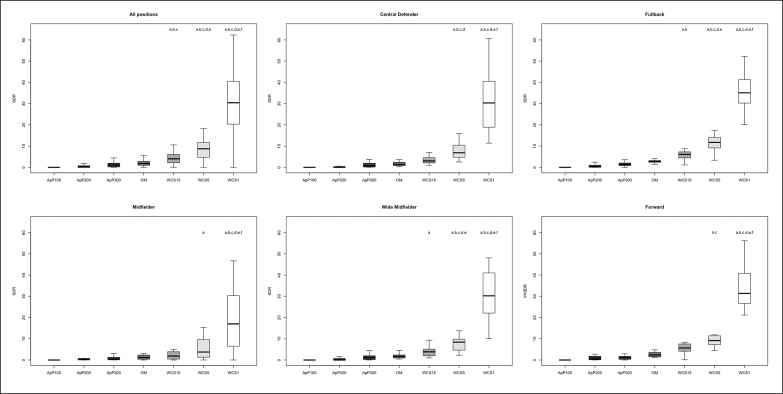
Significant differences between different WCS and various sided games considering sprint distance relative (SDR); a: significant differences compared to ApP100; b: significant differences compared to ApP200; c: significant differences compared to ApP300; d: significant differences compared to OM; e significant differences compared to WCS15; f: significant differences compared to WCS5.

The differences between playing positions and game type taking into consideration TDCR, HSDR, VHSDR, and SDR are outlined in Figures 1, 2, 3, and 4, respectively. The analysis showed interactions between game type and player positions (p < 0.001) for TDCR (Figure 1). Particularly, WCS1, WCS5, and WCS15 showed higher values than SSG formats for CD (p < 0.05; ES: 4.6–0.5), FB (p < 0.05;

ES: 7.5–1.1), MF (p < 0.05; ES: 13.9–0.5), WM (p < 0.05; ES: 5.2–1.1) and FW (p < 0.05; ES: 6.1–1.1). Furthermore, only WM (p < 0.05; ES: 6.5–1.4) and FW (p < 0.05; ES: 5.5–1.7) showed significantly greater values during WCS analysed compared to OM. Additionally, WM and CD SSGs played on ApP200 and ApP300 showed significantly higher (p < 0.001) TDCR values than ApP100. Figure 2 shows interactions between playing position and game type. All positions obtained higher (p < 0.005) HSDR values during all WCS compared to sided game formats, except for MF. For this position, no differences were found between WCS15 and ApP200, and ApP300 (p > 0.05). Figure 3 underlines the interactions between playing position and game type for VHSDR. Specifically, CD (p < 0.05; ES: 5.2–1.3), FB (p < 0.05; ES: 6.2–2.1), and WM (p < 0.05; ES: 4.9–1.4) positions showed higher VHSDR values during all WCS in comparison with all SSG formats, except for MF and FW. For these two positions, differences were not found between WCS15 and ApP200, and ApP300 (p > 0.05). Only WM exhibited significantly higher VHSDR values during OM compared to ApP100 (p < 0.05; ES: 2.5). Figure 4 shows the differences between WCS and sided games taking into consideration the SDR. Considering WCS1, all positions reported higher SDR values compared to all SSGs and OM. No differences were found between WCS15 and ApP200 and ApP300, except for FB and WM. Significantly higher SDR values were recorded in WCS15 compared to ApP100 for FB and WM (p < 0.05; ES: 3.2 and 2.0, respectively).

**FIG. 5:**
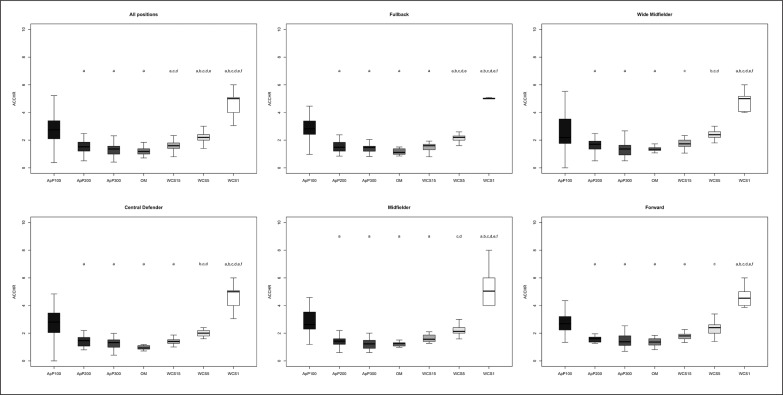
Significant differences between different WCS and various sided games considering number of total high-intensity accelerations relative (ACCHR); a: significant differences compared to ApP100; b: significant differences compared to ApP200; c: significant differences compared to ApP300; d: significant differences compared to OM; e significant differences compared to WCS15; f: significant differences compared to WCS5.

**FIG. 6:**
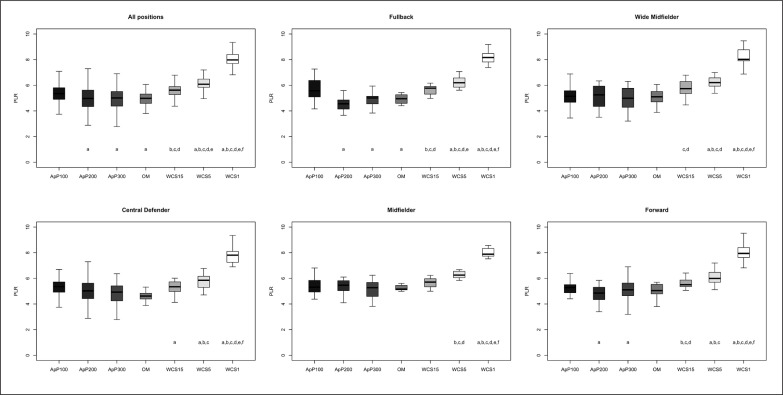
Significant differences between different WCS and various sided games considering player load relative (PLR); a: significant differences compared to ApP100; b: significant differences compared to ApP200; c: significant differences compared to ApP300; d: significant differences compared to OM; e significant differences compared to WCS15; f: significant differences compared to WCS5.

Mechanical values, ACCHR and PLR are shown in Figure 5 and Figure 6, respectively. ACCHR values were significantly greater during WCS1 compared to WCS5 (ES: 3.8) and WCS15 (ES: 4.6), OM (ES: 5.5), and all SSG formats (p < 0.001; ES: 5.1–2.1). ApP100 showed significantly higher ACCHR values compared to other SSG formats (ApP200 and ApP300) (p < 0.05; ES: 1.4 and 1.6, respectively), OM (p < 0.05; ES: 1.7) and WCS15 (p < 0.05; ES: 1.2) for all positions analysed separately. Interactions between playing positions and game type considering PLR are shown in Figure 4. WCS1 showed significantly greater PLR values compared to the other formats analysed for all positions considered (p < 0.001; ES: 5.4–3.3). FB and FW had higher PLR values during ApP100 in comparison with ApP200 (p < 0.05; ES: 1.6 and 0.8, respectively) and ApP300 (p < 0.05; ES: 1.2 and 0.3, respectively).

**FIG. 7:**
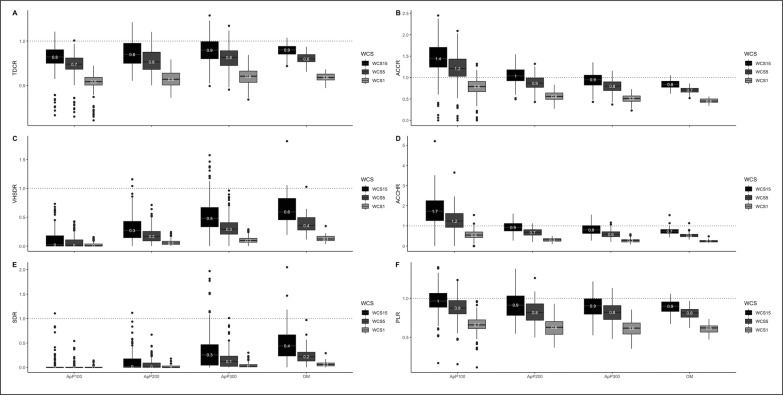
Various-sided games and official matches percentages (%) according to different worst-case scenarios for all players’ positions. ApP100: Area per player (<100 m^2^ · player^-1^); ApP200: area per player (ranged from 101 to 200 m^2^ · player^-1^); ApP300: area per player (ranged from 201 to 350 m^2^ · player^-1^). A): total distance covered relative (TDCR): B): total number of accelerations relative (ACCR); C): very high-speed distance relative (VHSDR); D): total number of high-intensity accelerations relative (ACCHR); E): sprint distance relative (SDR); F): player load relative (PLR)

Figure 7 shows the different SSG formats and OM, considering the relative load normalized across all three analyzed WCS for all players’ positions together. The ApP100 format represents, 50, 70, and 80% compared to WCS1, WCS5, and WCS15 respectively for TDCR. Taking into account VHSDR and SDR, ApP100 greatly underestimates the worst-case scenarios of match play (WCS1: 2 and 0%, WCS5: 7 and 2%, and WCS15: 11 and 3%, respectively). Considering mechanical values, the ApP100 format overestimated or reached similar values according to the worst-case scenarios for both ACCR (WCS1: 80%, WCS5: 120%, and WCS15: 140%) and ACCHR (WCS1: 50%, WCS5: 120%, and WCS15: 170%). The results obtained for the ApP200 format analysing TDCR were similar to what was observed in the ApP100 format. The ApP200 format represents 60, 80, and 80% compared to WCS1, WCS5, and WCS15 respectively for the above-mentioned variable. Considering high-intensity locomotor activities (HSDR and SDR), ApP200 did not exceed 30% compared to worst-case scenarios of match play (WCS1: 6 and 2%, WCS5: 20 and 7%, and WCS15: 30 and 10%, respectively). Regarding mechanical values, the ApP200 format reached values below the worst-case sce-narios of competition for both ACCR (WCS1: 60%, WCS5: 90% and WCS15: 100%) and ACCHR (WCS1: 30%, WCS5: 70%, and WCS15: 90%, respectively). The ApP300 format represents 60, 80, and 90% compared to WCS1, WCS5, and WCS15 respectively for TDCR. Taking into account VHSDR and SDR, ApP300 underestimates the worst-case scenarios of match play (WCS1: 10 and 4%, WCS5: 30 and 10%, and WCS15: 60 and 30%, respectively). Analysing mechanical values, the ApP300 format underestimated values according to the worst-case scenarios for both ACCR (WCS1: 50%, WCS5: 80%, and WCS15: 90%) and ACCHR (WCS1: 30%, WCS5: 60%, and WCS15: 80%).

## DISCUSSION

The main purpose of this study was to determine whether the physical performance of young soccer players during various SSGs underload, replicate, or overload the requirements of the worst-case scenarios (WCS) during match play considering external load measures. To the authors’ knowledge, no previous study has analysed the differences between WCS and SSG according to ApP with young soccer players. Our main findings were that WCS5 and WCS1 showed significantly higher values than SSG formats (ApP100, ApP200, and ApP300) and OM taking into account locomotor variables (TDCR, HSDR, VHSDR, and SDR). However, no significant differences were found between WCS15 and SSGs when sprint distances were analysed in most playing positions. Regarding mechanical (ACCHR) values, ApP100 showed significantly higher values than the WCS, except for WCS1.

There were differences between worst-case scenarios and ApP formats for the players depending on their field position. WCS1 and WCS5 were higher for TDCR values compared to other formats across all playing positions. However, considering WCS15, we found that TDCR was higher than the ApP200 and ApP300 for all positions, except for midfielders. Maybe the greater distance per minute reached in this position during both training sessions [26] and during SSG formats compared to other positions [27, 28] could explain these outcomes, since they are related to the positional role of linking defence and attack. These findings are in line with previous research [17, 20]. Therefore, these tasks seem less appropriate to replicate the most demanding phases of match play in the TDCR. Hence, SSGs with different ApP (ApP100, ApP200, and ApP300) reached 53, 57, and 60% respectively of TDCR of the WCS1. However, when using a bigger format (i.e., ApP300), the results showed similar values to different worst-case scenarios. Therefore, these tasks could be optimal to prepare TDCR worst-case scenarios of greater duration (i.e., WCS5 and WCS15).

In relation to high-intensity activities (i.e. HSDR, VHSDR), similar tendencies were found. HSDR and VHSDR were significantly higher in WCS1 and WCS5 compared to the different SSGs for all positions. However, no significant differences were found in HSDR and VHSDR between WCS15 and ApP200, and ApP300 for midfielders. A possible explanation for this fact could be that midfielders produce less HSDR and VHSDR during official matches [29]; hence, a lesser peak of 15 minutes was reached. In consequence, there are no differences between WCS15 and SSGs with larger area per player. Significant differences were found when we analysed the rest of the playing positions. FB and WM reached the greatest values during competitions in these variables; therefore, big differences exist between the most demanding passages and sided games. Regarding SDR values, WCS1 was higher than SSGs for all playing positions. Similarly, WCS5 was higher than SSGs for CD, FB, and WM, except for MF and FW. Once again, the shorter sprint distance reached for both positions during match play could be behind these outcomes. Additionally, no differences were found between WCS15 and SSGs for most positions. Hence, SSGs could be a good solution when the practitioners aim to prepare the most demanding passages of 15 minutes duration.

Current soccer presents more high-intensity actions and greater sprint distances [30], being decisive in both attacking and defensive soccer situations, and they are considered a key measure of physical performance in soccer [31, 32]. In contrast, one of the most used tasks during the training process is the SSGs [33]. However, the SDR and VHSDR observed during these types of drills were significantly lower than the demands observed in the worst-case scenarios of the competition taking into account different time periods in all playing positions. According to Dalen et al. [20], the discrepancy between SSGs and match peaks with respect to VHSDR and SDR could be due to several factors such as the size of the pitch during SSGs. The frequent use of smaller pitch size (ApP100 and ApP200) during the training sessions could be behind these differences. In addition, the reduced peak VHSDR and SDR recorded in ApP300 compared to WCS could potentially be due to the time constraints, pacing strategies, and psychological or motivational factors resulting in fewer opportunities to reach similar values to those found in the most demanding phases of match play [17, 34]. According to Riboli et al. [6], we would need more than 350 m^2^ · player^-1^ to replicate the official match peaks of high intensity and sprint distance in elite adult players. In consequence, we could mention that SSGs do not prepare players adequately for the most demanding phases of competition, specifically for WCS1 and WCS5, in relation to VHSDR and SDR. However, sided games with larger areas could be used to prepare WCS15 since no differences were found between WCS15 and SSG formats for each position analysed. Consequently, coaches and practitioners should consider the appropriate exposure of players to HSDR and SDR with the aim of either developing or maintaining their capacity to perform high-intensity efforts required frequently during WCS of match play [35]. Thus, the results of this study show that supplementary high-speed running drills should be planned and periodized suitably concurrently with SSGs to prepare for WCS for young soccer players [36, 37], specifically for positions with high demands of high-intensity and sprint distances (i.e. FB and WM). Additionally, this type of training could reduce the risk of non-contact ham-string injuries [38, 39, 40] and therefore should be considered in the practice.

Previous research concluded that accelerations and decelerations remained unchanged across different ApP used [6, 41]. Hence, no differences in accelerations and decelerations were found by Gaudino et al. [41] between SSGs with different areas per player. Conversely, we found that SSGs played on small areas (i.e., ApP100) had higher ACCHR values compared to WCS15 and WCS5 for all field positions, except for wide midfielders, where no differences were found. Wide midfielders reach the highest number of high-intensity accelerations during match play [16, 42]. Accordingly, similar values were observed between ApP100 and worst-case scenarios for this play position. However, it seems that SSG formats did not stim-ulate players sufficiently to cope with acceleration demands that occurred during WCS1 taking into consideration all play positions. We observed that players performed a higher number of both total and high-intensity accelerations per minute during ApP100 compared to WCS15 and WCS5. However, the greatest values were found during WCS1. Present data are in line with Dalen et al. [20], who observed that during SSG (4 vs 4) with professional soccer players, a higher number of ACCR was performed compared to the peak 5-minute intensity. Similarly, Martín-García et al. [16] also with professional Spanish soccer players reported that SSGs (i.e., 5 vs 5 and 6 vs 6) represented approximately 115% in relation to WCS5 for the ACCHR values. Nevertheless, neither research group reported data for WCS using time windows of shorter duration (i.e., WCS1). Hence, taking into account our results, it seems that when aiming to pre-pare players for the most demanding phases of 1-minute duration a supplementary task emphasizing ACCR and ACCHR values may be needed, since the ApP formats analysed represented a maximum of 78 and 56% respectively, compared to WCS1. However, if the aim is to prepare WCS with a longer time window, it seems that both ApP100 and ApP200 SSG formats may be suitable.

The present research has some limitations. First, more observations analysing ApP300 would be needed to reach more powerful conclusions. Secondly, the varying duration and training prescription between SSG formats is a limitation as this could have influenced the pacing strategies of players and it should be into account when the results are analysed. As another limitation, we only took into account the average values of SSGs without considering the most demanding phases during these drills. More research analysing the WCS and the SSGs’ peaks could elucidate this topic. Lately, internal load (i.e., heart rate and the rate of perceived exertion) were not examined. Hence, an aggregate analysis between internal and external load would provide more accurate information. However, the impossibility of collecting the rate of perceived exertion after each SSG format during the daily real-life training routine may limit the opportunity to monitor consistently the internal and perceived load.

## CONCLUSIONS

This study provides useful information for practitioners on the impact of SSG formats on physical load in relation to the WCS of competitive match play. The results highlight the importance of expressing the demands of the game formats relative to the WCS since we found that WCS5 and WCS1 were significantly higher than SSG formats (ApP100, ApP200, and ApP300) and OM taking into account locomotor variables (TDCR, HSDR, VHSDR, and SDR) and mechanical variables (ACCR, ACCHR, and PLR). In addition, larger SSG formats had greater TDCR and VHSDR values than smaller formats. However, when we analysed mechanical variables (ACCHR and PLR), we found that smaller SSGs had higher values compared to larger SSGs and OM. Only the ACCHR values exceed the values in the WCS5 and WCS15 during ApP100 formats, while the demands of other measures do not do so in any cases. It seems that if the objective is reaching ACCHR values like WCS, ApP100 formats could be valid. However, it may be necessary to design other types of tasks when practitioners aim to reach locomotor values (TDCR, HSDR, VHSDR, and SDR) similar to the most demanding passages of match play.

The present findings have several practical applications. In the first instance, to replicate the locomotor (TDCR, HSDR, and SDR) and mechanical values (ACCR, ACCHR, and PLR) reached during the WCS1 it seems that supplementary drills should be planned since the SSGs analysed did not cope with the physical demands of the most demanding phases of match play. Similarly, SSG formats with specific sprinting rules, individualized positional drills, transition-sided games, or running-based exercises seem to be needed when the objective is to reproduce VHSDR, and SDR values reached during WCS5. However, to prepare WCS15, sided games with larger areas could be a good choice since no significant differences were found between WCS15 and SSG formats for all positions analysed. The variables that have the lower percentage in relation to the most demanding phases of competition in all SSG formats studied, especially in the smallest formats of training games, are TDCR, VHSDR, and SDR. Lately, the different WCS during matches used in this study could be used as benchmarks to develop position-specific supplementary high-speed running training for elite young soccer players. This study provides useful information for coaches and practitioners on the impact of SSG formats on physical external load in relation to the WCS of competitive match play. The results highlight the importance of taking into account the most demanding passage of play to plan and periodize training load across the microcycle. Additionally, it seems important to compare match peaks with SSGs to assess the physical load imposed and include supplementary running drills if required.
